# Personality Traits and Impulsivity Tasks Among Substance Use Disorder Patients: Their Relations and Links With Retention in Treatment

**DOI:** 10.3389/fpsyt.2020.566240

**Published:** 2020-09-08

**Authors:** Jesús Gómez-Bujedo, Óscar M. Lozano, Pedro Juan Pérez-Moreno, José Andrés Lorca-Marín, Fermín Fernández-Calderón, Carmen Diaz-Batanero, Enrique Moraleda-Barreno

**Affiliations:** Department of Clinical and Experimental Psychology, University of Huelva, Huelva, Spain

**Keywords:** cognitive impulsivity, Diagnostic and Statistical Manual of Mental Disorders (Fifth Edition), personality traits, Personality Inventory for Diagnostic and Statistical Manual of Mental Disorders (Fifth Edition), substance use disorders, treatment adherence

## Abstract

**Background:**

Various authors have described the elements of impulsive approach and inhibitory control in drug users. These two components have been studied in terms of personality traits, performance on tasks that measure impulsive behavior, and neurophysiology. However, few studies have analyzed the association between these constructs. Thus, the aim of the present study is to analyze the associations between personality traits and performance on impulsivity tasks.

**Methods:**

A follow-up study was conducted with a baseline assessment at the beginning and end of treatment. The sample was composed of 121 patients undergoing treatment in therapeutic communities. Personality domains were evaluated through the PID-5. The impulsivity tasks employed were the Iowa Gambling Task (IGT), Delay Discounting Test (DDT), Go/No-Go and Stroop test.

**Results:**

A correlation was found between DDT scores and the domains of detachment (r = -.315; p<.01), antagonism (r = -.294; p<.01), and disinhibition (r = .215; p<.05). Performance on the Stroop task was significantly associated with psychoticism (r = .232; p<.05) and negative affect (r = .212; p<.05). Multivariate analysis revealed that IGT scores and negative affect predict retention in treatment.

**Conclusions:**

These findings partially support the hypothesized association between sensation-seeking personality traits and detachment with impulsive choice tasks; and the relationships between negative affect and psychoticism traits with performance on inhibitory control tasks. Further, impulsive choice task scores and negative affect are both shown to predict retention in treatment.

## Introduction

The association between personality traits and drug use is a widely studied topic. Until the advent of the DSM-III, substance use disorders were considered to be a subcategory of personality disorders (1). Today, the evidence is inconsistent regarding the existence of an “addictive personality” (2), although some findings suggest an association between certain personality domains and drug use. For example, low conscientiousness has been associated with the initiation of drug use in young people (3, 4), while high disinhibition, low conscientiousness, and aggressiveness have been associated with substance use disorder (5). In general terms, there is a consensus that the most impulsive personality traits, and emotional instability, are linked with the appearance of drug-using behaviors. Moreover, substance addiction is often associated with specific impaired Response Inhibition and Salience Attribution (iRISA) networks in addition to comorbidity with other disorders (6, 7).

Impulsivity is a complex and multifaceted trait, which has been traditionally evaluated through a multi-method approach that distinguishes between self-report and behavioral tasks. Self-reports are based on questionnaires that ask people about their behavior patterns in certain contexts and are generally the most frequently used methods for studying impulsive personality traits (8). In behavioral tasks, certain performance indicators are used to infer impulsive behavior (e.g., reaction times, alternative choice tasks). These, in turn, can be categorized into impulsive action (response inhibition, inhibitory control), characterized by the inability to stop a prepotent response that the subject might naturally emit, and impulsive choice (impulsive decision making), which is related to the difficulties in optimizing the decision-making process. Further, impulsive action can be differentiated into motor inhibition (related to the ability to inhibit an automated response), and interference control (a cognitive form of inhibition, which involves the suppression of competitive and distracting information to maintain response performance). Broadly speaking, impulsive choice can be evaluated by the delay discounting task, which measures the change in the subjective value of a reward according to the time it takes to be released, and impulsive decision making, which measures decision making under conditions of ambiguity (9, 10).

At the trait level, the associations between personality domains, facets of impulsivity, and drug use have been well documented (11, 12). Evidence has also been reported in favor of an association between performance on impulsivity tasks and drug use (13–16). However, the associations between performance on neuropsychological impulsivity tasks and the five-factor model personality traits have been less widely studied. In community samples, scores on impulsive choice tasks such as the Iowa Gambling Task (IGT) have been found to be associated with the domains of extraversion, neuroticism, and openness (17–20). Buelow and Cayton (21) also reported a correlation between IGT scores and agreeableness, while other authors have found no association between performance on this behavioral task and any personality trait (22, 23). In delay discounting tasks (DDT), poor performance has been associated with low levels of openness and conscientiousness, and high extraversion and neuroticism (24–26). Studies of impulsivity suggest that extraversion influences execution time on the Stroop task, with a positive correlation between the two constructs (27–29), while no association has been found with neuroticism, agreeableness, and conscientiousness. Similarly, other authors have found no association between Stroop scores and personality domains (30). In studies conducted with drug users and healthy volunteers, Ersche et al. (31) found a correlation between performance on executive function tasks and certain facets linked to emotional functioning, impulsive-compulsive, and self-evaluation traits. In subjects with alcohol dependence, Tomassini et al. (32) found that IGT scores were associated with the novelty-seeking and persistence domains of Cloninger’s model of personality.

For some authors, the relationships between impulsive personality traits and the execution of impulsivity tasks (in addition to neurophysiological processes) must be related to each other, given that the two processes underlie addiction (33). In particular, in addiction there is a process associated with the propensity or urge to approach drugs; while it is also possible to identify a process associated with a reduced capacity to inhibit this approach behavior. According to this model, the impulsive approach component includes personality domains such as sensitivity to reward, sensation-seeking or extraversion, together with scores on impulsive choice and delay discounting tasks. The inhibitory control component includes personality traits such as constraint and scores on behavioral tasks related to impulsive action or motor disinhibition (33). However, Gullo and Potenza (34) have indicated the need to analyze data from multiple impulsivity measures simultaneously in order to better understand these processes.

In addition, Section III of the Diagnostic and Statistical Manual of Mental Disorders (Fifth Edition) proposes an alternative diagnostic model for personality disorders based on the identification of pathological personality facets. This model allows both the diagnosis of personality disorders and the establishment of clinical profiles in addition to being equivalent to other personality models such as the five-factor model (FFM) (35). Further, previous studies have demonstrated an association between AMPD and the UPPS-P model of impulsivity (36) and its usefulness in identifying the profiles of drug-using patients (37). Therefore, within the field of addiction, this is a useful personality model, from both a clinical and research standpoint.

Thus, the present study had the following objectives:

- To analyze the correlation between personality traits according to AMPD and scores on impulsivity tasks in a sample of patients with substance dependence. Following the proposal of Gullo et al. (33), we expected to find an association between performance on impulsive choice tasks and those personality traits that are most strongly linked to disinhibition, extraversion, and sensation-seeking, while performance on motor impulsivity tasks will be more strongly associated with negative-affect-related personality traits.
- To analyze the predictive capacity of personality traits and performance on impulsivity tasks with regard to treatment retention. According to previous studies, patients that show traits and behaviors more strongly linked to sensation-seeking are less likely to remain in treatment.

## Method

### Participants

The sample was composed of 121 inpatients (91.7% males) with a mean age of 37.94 (SD = 10.23) years. The patients were being treated in three public therapeutic communities (TC) located in Andalusia (Spain). These patients were referred from outpatient addiction treatment centers (ATCs). The primary reasons for referral are: i) the inability to maintain abstinence during outpatient treatment; and, ii) the need for intensive and continuous treatment. In all three TCs, patients receive cognitive behavioral treatment for addictions, which is standardized according to a single protocol. This treatment program is multidisciplinary and includes medical and psychological care, as well as health care and participation in educational programs.

A total of 72.7% of the participants had been diagnosed with cocaine use disorder (CUD) according to DSM-IV criteria, 49.6% with alcohol use disorder, 37.2% with cannabis use disorder and 27.3% with heroin use disorder. Of the sample, 58.3% had been diagnosed with more than one substance use disorder. During the month prior to admission to the TC, 42.1% reported using cocaine, 35.5% alcohol, 33.1% cannabis, and 16.5% heroin.

To participate in the study, patients had to meet the following inclusion criteria: 1) No vision problems or other physical impairments that would hinder the completion of computerized tests; 2) the ability to read and write; 3) the absence of comorbid mental disorders; 4) be of legal age; and, 5) sign the informed consent form.

With regard to education levels, 63.7% had completed primary education, 35.5% secondary education, and 15.7% had completed university studies. Most of the participants (59.5%) were unemployed at the time of entering the therapeutic community, 22.3% were working, and the remaining percentage were retired. In terms of marital status, 10.7% were married, 65.3% were single, and the remainder were divorced.

After 1 year of assessment, a total of 38 patients withdrew from their therapeutic process while 83 patients successfully completed the therapeutic objectives of the TC. The former spent a mean of 87.97 (SD = 57.29) days in treatment and the latter a mean of 202.77 (SD = 75.82) days, these differences being statistically significant (t_93,353_ = 9.177; p<.01).

### Instruments

We used the following impulsive decision-making tasks, which are measures related to the impulsive approach component:

- *Delay Discounting* (38). This questionnaire is composed of a fixed set of 27 options and consists of monetary choice in which participants are asked about their individual preferences when choosing between small but immediate rewards and greater but delayed rewards. In this study we used, as an index of impulsivity, the area under the curve [AUC; (39)]. A higher score on this index is taken to indicate lower impulsivity.
- *Iowa Gambling Task* (40). This is a classic task for evaluating decision-making under conditions of ambiguity. Participants must choose between four decks of different cards, which have different reinforcement and punishment schedules. The task is to choose cards in order to gain the maximum or greatest benefits, although in the long run these produce losses. The computerized version was employed (41), using the total score as the index of impulsive decision-making. A higher score is associated with more adaptive decision-making.

In order to evaluate inhibitory control, we employed the following tasks:

- *Inhibition—Affective Go/No-Go* (42). In this task, participants were required to press a key when a Go stimulus (one letter) appeared on the screen (80% of the trials), and to not press the key when the No-Go stimulus appeared on the screen (a different letter; 20% of the trials). Participants received feedback on their successes and errors through audio signals. The task consisted of two blocks of 60 trials. The first block was administered after neutral images had been displayed, while the second block was administered after viewing positive affective images. All images were extracted from the International Affective Picture System [IAPS; (43)]. The index of impulsivity in this task was the number of commission errors (i.e. responses to No-Go stimuli) in the neutral and positive blocks. A higher number of commission errors is taken to indicate poor inhibitory control.
- *Inattention: Stroop Task* (44). The Stroop task can also be considered as a measure of response inhibition (45). This task generates interference between the reading of words and the naming of colors (Stroop interference effect), so that the reaction time varies depending on the congruence or incongruence between the word and the color. The participants are required to inhibit the reading of the written word and respond with the color in which it is written. The index of impulsivity in this task is the Stroop Interference score. Increased interference is associated with increased motor impulsivity.
- *Substance Dependence Severity Scale* (46). The SDSS is a semi-structured interview that evaluates the severity of substance dependence. In this study, we assessed the severity of dependence on cocaine, alcohol, cannabis and opiates using the Spanish version adapted to the DSM-5 criteria (47). Reliability, estimated by internal consistency, was higher than.83 for all substances. A higher score is taken to indicate a higher severity of dependence.
- *Personality Inventory for DSM-5 Short Form* [PID-5SF; (48)]. We administered the Spanish version of this instrument (49, 50). This instrument originates from the DSM-5 Alternative Model for Personality Disorders [AMPD; (51)] and evaluates the 25 established facets by organizing them into the following five personality domains: negative affect, detachment, antagonism, disinhibition, and psychoticism. These personality domains are considered to be the maladaptive extremes of the five-factor model (52). This instrument has shown adequate psychometric properties in terms of reliability and evidence of validity for its use with patients that consume drugs (50, 53).

### Procedure

The interviews were conducted by a psychologist with experience in patient assessment, who had been specifically trained to administer these instruments. The interviews were conducted in individual sessions at the centers where the patients were receiving their treatment.

Initially, the therapists informed patients that researchers at the University of Huelva were conducting a study. The therapists specified that their decision to participate (or not) in the study would have no effect on their therapeutic process. If patients agreed to participate, they were then sent to the interviewer.

Prior to administering the tests, the psychologist informed the patients of the objectives of the study and indicated the need to collect data related to their retention/dropout in treatment during the year following the interview. The psychologist also informed patients of their rights during participation and resolved any concerns before the start of the study. Once written informed consent had been given, the administration of the tests began. Initially the psychologist administered the SDSS to the patients. The patients then individually performed the behavioral tasks on a computer. Before starting each test, the interviewer explained the instructions to the patients, and made sure that they had understood them. Before each task, the patients carried out a test. Finally, the psychologist also administered the PID-5 to the patients. The tests lasted approximately 45‑60 min.

This study has been approved by the ethics committee of the University of Huelva.

### Analysis

The scores were initially checked for normality using the Shapiro-Wilks test, which confirmed that none of the measures of impulsive behavior followed a normal distribution (W_DDT_ = 0.93, z = 4.160, p = .000; W_IGT_ = 0.95, z = 3.417, p = .000; _Stroop_ = 0.92, z = 4.554, p = .000; W_Go/no-Go_ = 0.99, z = 1.974, p = .021). We therefore decided to use non-parametric tests to test the hypotheses (54). The relationships between the quantitative variables were analyzed using Spearman’s Rho correlation coefficient. A Mann-Whitney U test was employed to analyze the correlation between each quantitative variable (i.e. each of the personality traits) and the type of discharge from the TC.

To analyze the predictive capacity of the variables with regard to treatment retention, the Kernel-Based Regularized Least Squares [KRLS; (55)] was used, which allows for conducting a regression analysis without relying on assumptions regarding data distribution. For this, KRLS estimator learns the functional form from the data and thereby protects inferences against misspecification bias. The interpretation and inferences made on the basis of these coefficients are similar to those provided by standard regression models. Two KRLS analyses were conducted in this study. The first included time in treatment as a dependent variable, while the second used retention in treatment as a dependent variable (a value of 0 indicates abandonment of treatment; and a value of 1 indicates discharge from treatment). The independent variables of these analyses were the PID domains and the behavioral measures of impulsivity. Age, gender, and severity of alcohol and cocaine dependence were also introduced into the model in order to control for the effects of these variables.

All analyses were conducted using Stata Vers 14.2 software.

## Results

### Associations Between PID Domains and Behavioral Measures of Impulsivity

**Table 1** shows the associations between measures of impulsive choice, motor impulsivity and personality domains. It can be observed that DDT scores are negatively correlated with the domains of detachment, antagonism and disinhibition. This indicates that these personality domains are associated with a more impulsive pattern of decision-making. Among the motor impulsivity tasks, Stroop scores show a statistically significant association with the psychoticism and negative affect domains. Specifically, greater interference is associated with a higher number of traits linked to negative affect and psychoticism. When the correlation analyses were adjusted for multiple comparisons, statistically significant associations were observed between DDT scores and both detachment (r = -.318; p = .017) and antagonism (r = -.294; p = .04).

**Table 1:** Correlations between personality domains and scores on impulsivity tasks.

	Negative affect	Detachment	Antagonism	Disinhibition	Psychoticism
DDT	-.153 (p = .095)	-.315 (p = .001)	-.294 (p = .001)	-.216 (p = .019)	-.134 (p = .149)
IGT	.035 (p = .704)	.040 (p = .667)	-.090 (p = .335)	.034 (p = 711)	.142 (p = .125)
Stroop	.232 (p =.011)	-.090 (p = .670)	.014 (p = .877)	.125 (p = 176)	.212 (p = .021)
Go/No-Go	-.031 (r = .739)	-.027 (r = .769)	-.073 (r = .431)	-.029 (r = .760)	-.010 (r = .912)

### PID and Impulsivity Tasks: Association With Severity of Dependence

Analysis of the association between personality domains and dependence severity revealed significant positive correlations between the severity of cocaine dependence and the traits of negative affect (r = .192; p = .037), antagonism (r = .224; p = .015), disinhibition (r = .216; p = .019), and psychoticism (r = .216; p = .019). Among the impulsivity tasks, it is observed that DDT scores correlate negatively with the severity of cocaine dependence (r = -.267; p = .003), such that greater severity is associated with greater impulsivity. It was also observed that the severity of alcohol dependence is significantly correlated with the Go/no-Go false alarm rate (r = .253; p = .006). Correlation analysis adjusted for multiple comparisons revealed that the DDT scores were associated with the severity of cocaine dependence (r = -.282; p = .031).

### PID and Impulsivity Tasks: Associations With Treatment Retention

It was observed that time in treatment was negatively correlated with traits of negative affect (r = -.195; p<.05), antagonism (r = -.232; p<.05), and disinhibition (r = -.205; p<.05). When applying Bonferroni’s correction for multiple tests, no statistically significant associations were found between the above variables. The KRLS model, controlling for age, gender and severity of alcohol and cocaine dependence, revealed that the negative affect and IGT coefficients were statistically significant (**Table 2**).

**Table 2:** KRLS analysis for the prediction of treatment retention (days in treatment).

	Avg.	SE	t	P<|t|	P25	P50	P75
Gender (female)	-1.809	2.375	-0.762	.448	-3.021	-1.768	-0.617
Age	0.021	0.031	0.665	.507	0.003	0.023	0.041
Severity of alcohol dependence	-0.023	0.025	-0.933	0353	-0.038	-0.024	-0.007
Severity of cocaine dependence	-0.008	0.020	-0.418	.677	-0.016	-0.006	0.002
Negative affect	-1.051	0.486	-2.161	.033	-1.552	-1.021	-0.577
Detachment	-0.001	0.478	-0.002	.998	-0.241	-0.005	0.182
Antagonism	-0.802	0.441	-1.821	.071	-1.103	-0.867	-0.491
Disinhibition	0.158	0.419	0.379	.705	-0.040	0.138	0.433
Psychoticism	-0.579	0.495	-1.171	.244	-0.878	-0.609	-0.223
DDT	-1.137	1.011	-1.124	.264	-1.771	-1.337	-0.627
IGT	-0.027	0.013	-2.119	.036	-0.037	-0.029	-0.017
Stroop	-0.003	0.005	-0.620	.537	-0.005	-0.003	-0.000
Go/No-Go	-5.700	7.986	-0.714	.477	-10.060	-5.776	-1.493

P25: 1^st^ quartile 25; P50: Media; P75: 3^rd^ quartile.

Analysis of dropout/discharge from therapy with respect to the study variables revealed that those who dropped out scored higher on negative affect and psychoticism (**Table 3**). KRLS analysis confirmed that none of the variables analyzed show predictive capacity with respect to the type of discharge.

**Table 3:** Relationships between type of discharge, personality domains, and scores on impulsivity tasks.

	Dropout [Mean (SD)]	Therapeutic discharge [Mean (SD)]	Mann-Whitney U	z	p	Effect size
Negative affect	1.89 (0.56)	1.64 (0.63)	1012.5	2.822	.005	0.26
Detachment	1.12 (0.59)	0.96 (0.67)	1356.5	0.825	.410	0.08
Antagonism	0.90 (0.63)	0.88 (0.69)	1160.5	1.963	.050	0.18
Disinhibition	1.70 (0.71)	1.48 (0.68)	1309.0	1.101	.271	0.10
Psychoticism	1.17 (0.60)	1.01 (0.60)	1129.5	2.143	.032	0.20
Stroop	0.10 (0.06)	0.09 (0.07)	1412.0	0.502	.616	0.05
Go/No-Go	0.06 (0.03)	0.07 (0.04)	1318.0	0.833	.405	0.08
DDT	0.44 (0.33)	0.43 (0.29)	1496.0	0.225	.822	0.02
IGT	2.37 (31.87)	3.90 (20.81)	1497.0	0.009	.993	0.00

## Discussion

The objectives of this study were: i) to analyze the relationships between behavioral measures of impulsivity and personality domains; and, ii) to analyze the correlation between these variables and retention in treatment and type of discharge. As mentioned previously, very few studies have addressed the relationships between performance on neuropsychological impulsivity tasks and the big five personality traits—and even fewer have focused on the pathological domains and facets of AMPD. With regard to the second objective, Foulds et al. (56), in their review, indicate the need to study the association between personality patterns and retention in treatment, given the clinical significance of this information. Furthermore, these objectives have been addressed for the first time from the alternative model of personality disorders (AMPD) proposed in DSM-5.

The results related to our first objective partially support the hypothesis. We observed that more impulsive scores on DDT are associated with the domains of detachment (associated with introversion in the FFM) and disinhibition (low conscientiousness in the FFM), these traits and behaviors being associated with the impulse to approach drugs (33). In addition, the association with conscientiousness has also been found in studies conducted with community samples (26, 57). Although not hypothesized, a relationship has been found between DDT scores and antagonism domain scores. One possible explanation for this relationship could be the high comorbidity between SUD and antisocial personality disorder (ASPD) (58, 59), of which antagonism is a characteristic domain (51). Moreover, an association has been reported between ASPD and performance on the DDT (60, 61). Additional research is needed to confirm these relationships. Further, the absence of the expected correlation between disinhibition and IGT scores could be due to the fact that tasks with a poorly defined probability of success (under ambiguous conditions) are not influenced by disinhibitory personality characteristics, unlike those with explicit outcomes and probabilities (under risk conditions) (22).

The association between negative affect and Stroop performance was also anticipated from a theoretical perspective (33). This association could be due to the role played by the amygdala in mediating emotional interference in tasks that require cognitive resources, with studies showing that patients with a high degree of neuroticism (associated with negative affectivity) show excessive activation of this area (62). Nor is it surprising that scores on psychoticism are correlated with Stroop performance, since this domain includes facets related to the impairment of cognitive processes. This association has been found in other studies with people that tend to show unusual beliefs, high creativity, and disorganized personality (63, 64).

With respect to the second objective, it was hypothesized that traits associated with sensation-seeking would reduce treatment retention, while those associated with cooperation and persistence would improve retention (56). Thus, it might be expected that both the disinhibition domain and scores on impulsive choice tasks would play a central role in predicting retention in treatment. Consistent with previous studies, TGI scores were shown to be a predictor of treatment retention (65–67). Among the personality traits, negative affect, rather than disinhibition, emerged as a predictor of treatment retention. The fact that—contrary to our original hypothesis—negative affect appears to be a predictor of retention, could be related to the fact that scores on this personality domain involve the assessment of negative experiences and emotions (e.g., anxiety, depression, emotional lability, and hostility). Such emotions could appear in patients with addiction resulting from adaptations in the extended amygdala circuits (68). Thus, in this case, negative affectivity could be measuring the state of the patients rather than their personality traits, as some authors have pointed out when using the PID in addiction patients (50).

Although this study provides results of interest for both the investigation and clinical management of these patients, it is necessary to bear in mind a series of limitations. Possibly the most notable of these is the (low) proportion of women in our sample. Although this gender bias is an accurate reflection of the percentage of women in treatment in this study setting (69), our findings should not be generalized to women, given the differences noted by some authors in impulsivity and personality patterns. Moreover, our study was conducted with a sample of 121 patients. A larger sample size would have been desirable to improve the statistical power of the study. However, it should be noted that these types of studies are generally conducted with small sample sizes—smaller than the one used here (70)—which reflects the complexity of conducting such studies. It is also necessary to take into account that the findings obtained in this work are mostly of a correlational nature, which prevents the establishment of causality between variables. Moreover, this is a transversal study, so in order to reach definitive conclusions it would be appropriate to carry out further longitudinal studies. It is also worth noting that in the literature the relationships between self-report and behavioral measures of impulsivity are usually low, which seems to indicate that they evaluate different constructs with little shared variance (71) and that attempts to find evidence for the integration of these constructs could be unsuccessful (11). Some authors claim that the confounding impact of methodological variations makes it difficult to integrate data from both types of measurement (10). Although this could be considered a limitation of the present study, it is also important to consider that both types of measures predict addiction-related behaviors (11) and that knowledge of how they are related could improve our understanding of these phenomena. As proposed by Stevens et al. (10) it would be desirable for future investigations to include assessment systems based on laboratory and self-report tests of different impulsivity constructs in order to create a true multi-trait-multimethod approach.

In spite of the limitations just mentioned, we consider that our findings contribute towards gaining a deeper understanding of the role of personality and impulsivity in addiction. Moreover, our results could contribute towards identifying patient profiles that could benefit from tailored intervention strategies that could help to improve the treatment outcomes of these patients.

## Data Availability Statement

The raw data supporting the conclusions of this article will be made available by the authors, without undue reservation.

## Ethics Statement

The studies involving human participants were reviewed and approved by the ethics committee of the University of Huelva. The patients/participants provided their written informed consent to participate in this study.

## Author Contributions

JG-B and ÓL designed the study. EM-B wrote the first draft of the manuscript. FF-C and CD-B have been implicated in data analysis. JL-M and PP-M conducted literature searches and completed the *Introduction* section of the paper. All authors contributed to the article and approved the submitted versión.

## Funding

This study was supported by the “Longitudinal study about the effect of treatment on executive function recovery in patients with cocaine and alcohol dependence: implications on treatment outcomes “provided by the Delegación del Gobierno para el Plan Nacional sobre Drogas (Spain) (grant number Q7150008F-2016/034)”.

## Conflict of Interest

The authors declare that the research was conducted in the absence of any commercial or financial relationships that could be construed as a potential conflict of interest.
